# Modeling Clinical States and Metabolic Rhythms in Bioarcheology

**DOI:** 10.1155/2015/818724

**Published:** 2015-08-06

**Authors:** Clifford Qualls, Raffaella Bianucci, Michael N. Spilde, Genevieve Phillips, Cecilia Wu, Otto Appenzeller

**Affiliations:** ^1^Health Sciences Center, University of New Mexico, Albuquerque, NM, USA; ^2^Department of Biosciences, Centre for Ecological and Evolutionary Synthesis (CEES), University of Oslo, Oslo, Norway; ^3^Department of Public Health and Pediatric Sciences, University of Turin, Turin, Italy; ^4^Department of Earth and Planetary Sciences, Institute of Meteoritics, University of New Mexico, Albuquerque, NM, USA; ^5^Cancer Research and Treatment, Center Fluorescence Microscopy Facility, University of New Mexico, Albuquerque, NM, USA; ^6^Department of Pathology, University of New Mexico, Albuquerque, NM, USA; ^7^New Mexico Health Enhancement and Marathon Clinics Research Foundation, Albuquerque, NM, USA

## Abstract

Bioarcheology is cross disciplinary research encompassing the study of human remains. However, life's activities have, up till now, eluded bioarcheological investigation. We hypothesized that growth lines in hair might archive the biologic rhythms, growth rate, and metabolism during life. Computational modeling predicted the physical appearance, derived from hair growth rate, biologic rhythms, and mental state for human remains from the Roman period. The width of repeat growth intervals (RI's) on the hair, shown by confocal microscopy, allowed computation of time series of periodicities of the RI's to model growth rates of the hairs. Our results are based on four hairs from controls yielding 212 data points and the RI's of six cropped hairs from Zweeloo woman's scalp yielding 504 data points. Hair growth was, ten times faster than normal consistent with hypertrichosis. Cantú syndrome consists of hypertrichosis, dyschondrosteosis, short stature, and cardiomegaly. Sympathetic activation and enhanced metabolic state suggesting arousal was also present. Two-photon microscopy visualized preserved portions of autonomic nerve fibers surrounding the hair bulb. Scanning electron microscopy found evidence that a knife was used to cut the hair three to five days before death. Thus computational modeling enabled the elucidation of life's activities 2000 years after death in this individual with Cantu syndrome. This may have implications for archeology and forensic sciences.

## 1. Introduction

“Zweeloo woman,” now held in the Drents Museum in Assen, the Netherlands, was discovered in 1951 in a bog and exhumed in the presence of an archeologist and a paleobotanist. Subsequent studies of her remains showed that she was of short stature and affected by bony abnormalities consistent with Léri-Weill dyschondrosteosis [1]. Her scalp hair, when examined 2000 years after her death, was found to have been crudely cropped [1]. We found she also suffered from hypertrichosis, an excessive hair growth over her entire body including the face [2].

Dyschondrosteosis together with hypertrichosis and cardiomegaly, an enlarged heart, is characteristics of a recently described genetic disorder called Cantú syndrome (CS) [3].

Fifteen CS cases have been described, the majority of Mexican-Mestizo descent. The condition is suspected to be dominantly inherited [3].

Here we use modern histological methods and computational modeling, applied to scalp hair. The results give insights into her clinical and mental state for a few days before her death ~2000 year ago and may shed light on her burial in a bog instead of the customary cremation and interment in a cemetery.

We used modeling to show that the neuroautonomic control of biologic rhythms, metabolism, and behavior can be deduced from ancient material such as hair.

Thus our aim of determining life's activities from archived remains has been validated by widely applicable computational methods.

## 2. Materials and Methods

The Drents Museum in Assen, the Netherlands, donated the tissues obtained from specimen #1957/XII-13. Vincent van Vilsteren, curator of the Drents Museums of Assen, the Netherlands, gave permission to study the material. The New Mexico Health Enhancement and Marathon Clinics Research Foundation (NMHEMC, Research Foundation) in Albuquerque NM, USA, carried out the analyses. The institutional review board of the NMHEMC Research Foundation approved the study. All necessary permits were obtained for the described study, which complied with all relevant regulations.

### 2.1. Scalp and Scalp Hair

A small piece ~2 × 5 cm of brownish skin with reddish appearing hairs of varying length, in situ, was received for analysis. This was labeled “scalp skin of Zweeloo woman” without further description. Hair retrieved from bodies interred in bogs is usually, though not invariably, of a reddish discoloration because of the acidic environment of the bog.

### 2.2. Two-Photon and Confocal Laser Scanning Microscopy

We could not apply fluorescent stains to the tissue because of chemical changes in the skin-proteins (taphonomic changes) caused by the long immersion in the acidic bog.

**Figure 1:**
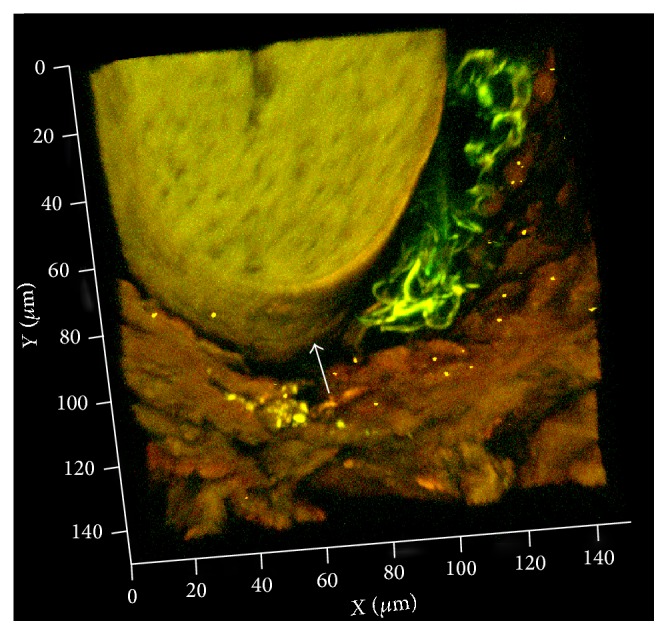
Three-dimensional image of the bog body's scalp skin by two-photon laser scanning microscopy. Unstained image (250 *μ*m thick) of an autofluorescent hair shaft with remnants of autofluorescent autonomic fibers in green adjacent to the hair root (arrow).

Therefore, standard methods were used to visualize unstained images (250 *μ*m thick or single hairs) (Figures 1 and 4).

### 2.3. Scanning Electron Microscopy

**Figure 2:**
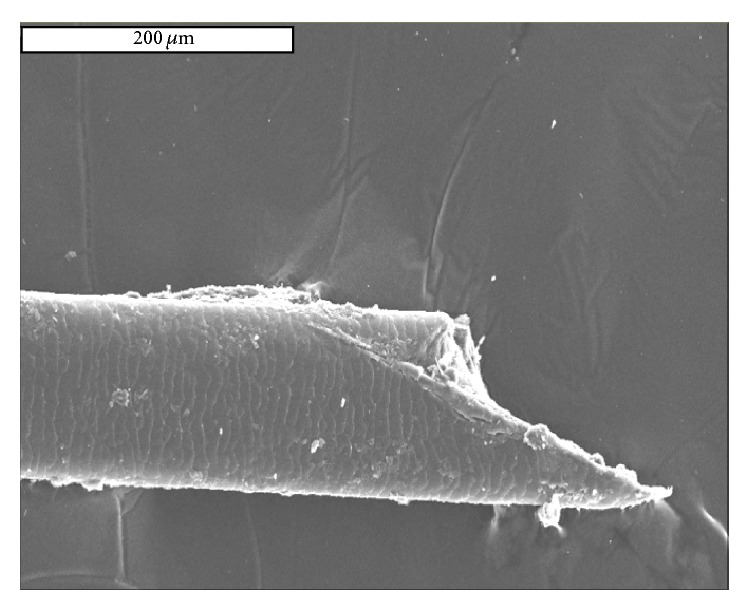
The end of a single scalp hair. Scissors would cut vertically across the hair; however, the shape of the cut suggests that this was made by a knife rather than scissors (scale bar in white above, 200 *μ*m).

**Figure 3:**
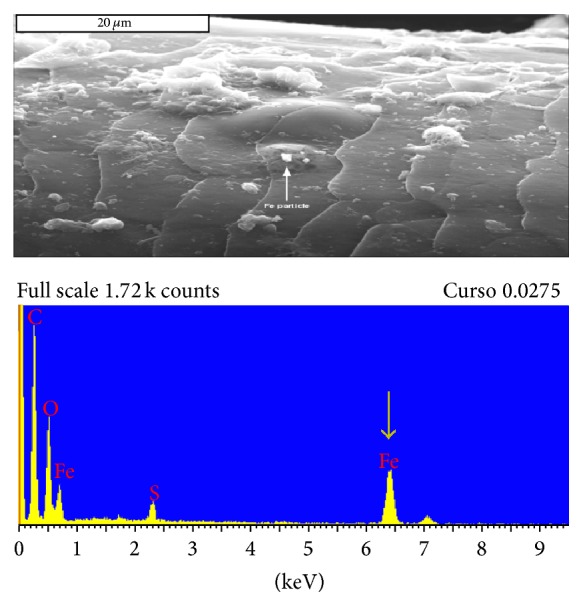
Particle (arrow) SEM image. This is the putative remnant of the hair cutting instrument, made of iron. Below, EDX of the particle showing the Fe peak of the spectrum (arrow).

The hair samples were coated with gold to provide better conductivity. A JEOL m 5800 electron microprobe (Figures 2 and 3) was used.

### 2.4. Biologic Rhythms

Our results were based on measuring the RI's of four hairs from controls yielding 212 data points and the RI's of six cropped hairs from Zweeloo woman's scalp yielding 504 data points.

The cropped hair remnants on the scalp skin were 10.4 mm, 9.1 mm, 11.4 mm, 12.2 mm, 17.9 mm, and 16.8 mm long.

### 2.5. Power Spectral Analysis of Repeat Intervals

We computed periodicities from the spectrum (periodogram) of a time series as a function of linear axis, such as, length along a strand of hair. We used growth rates in these measures to express frequency peaks in the spectrum as periodicities in units of days.

Determination of a high frequency peak is usually clear from the spectrum. However, determination of a low frequency peak can take several forms.

In the time series of RI intervals (averaging 0.132 mm for short/medium hairs RI size = 0.214 mm for the longer hair remnants), there is a high frequency peak at 0.32 radians/RI in each series (see Figure 5(b)). The periodicity of this sinusoid cannot be 52 weeks, since the average long hair remnant length of 17.4 mm would represent 6.5 annual cycles with an improbable annual growth of only 3 mm per year (proof by contradiction).

The low frequency spectral peak for the Zweeloo long hair is at 0.045 radians/RI, which is a factor of 7 lower than the high frequency. Therefore, the high frequency represents a daily periodicity and the low frequency represents a weekly periodicity.

An identified spectral peak (i.e., a daily or annual cycle) can be used to compute hair growth rate; hence we then have an absolute time scale. The growth rate based on her longer hair remnant is estimated as (see formula below in legend to Figure 5) 153 cm/year ~10x the normal hair growth rate of 16 cm/year. Consider

(1) Growth  Rate =dtfreqPeriod =0.214 mm/obs0.32  radians/obs1  day/cycle⁡2π  radianscycle⁡ =4.2 mm/day,mmmmmmmmwhere  each  observation=a  scale,mmmmmmmmmwhich  averaged=0.214 mm/obs.

This formula is derived from [4]. The corresponding annual growth of 153 cm/year is ~10 times the average annual growth rate of 16 cm/year, consistent with hypertrichosis.

The spectra were computed using finite Fourier transform which decomposed time series into sums of sine and cosine waves of varying amplitudes and wave lengths. We used PROC SPECTRA from SAS version 9.3 for the statistical computations (Figures 6, 7, and 8).

The low frequency/high frequency (LFHF) ratios were significantly increased (*P* < 0.02) and the low frequency (LF) variance significantly decreased (*P* = 0.02) consistent with an increased sympathetic drive to metabolism and also increased heart rate and stress during life.

### 2.6. Approximate Entropy (ApEn)

To validate statistically our results we used approximate entropy (ApEn) [5]. This analysis can quantify the degree of regularity and unpredictability in the fluctuations of time series data such as those used in this study.

Here we used *m* = 2 and *m* = 3 windows. We found that values of 80% discriminated best for this analysis between controls and Zweeloo woman. The ApEn in Zweeloo woman was higher (Figure 8) thus her ANS was functioning well and reflected the robustness of her ANS function consistent also with sympathetic activation.

A typical case of hypertrichosis is illustrated in Figure 9. This shows why such excessive hair growth can be emotionally devastating leading to stress induced sympathetic activation and tachycardia as we surmise was present in Zweeloo woman before her death.

## 3. Results

Standard histological examination of the scalp was not possible due to protein degradation after 2000 years in a bog. However, using two-photon microscopy on 250 *μ*m sections, we found preserved structures that appear to be hair and autonomic nerve fibers using the intrinsic fluorescence within the sample (Figure 1).

We then used the scanning electron microscope to visualize the end of her cut hair. This gave hints about the type of instrument used in her “hair cut” just before her death (Figure 2).

During the Roman period scissors and knives were made from either iron or copper [6]. We identify a particle on her hair by scanning electron microscopy, presumably, left from the “hair cut”; this was made of iron (Figure 3).

Energy-dispersive X-ray spectroscopy (EDX) is an accepted analytical tool used for elemental analysis applied here using the high-energy electron beam of the scanning electron microscope (SEM) used on the hair. We found iron particles on the surface of the hair (Figure 3), perhaps left by the cutting instrument. Additionally, this method of analysis revealed particles of the rare element, hafnium, which is, however, common in Dutch soil (not shown). Hafnium has also been found in hair and finger nail clipping of contemporaneous Scandinavian and Dutch people [7].

**Figure 4:**
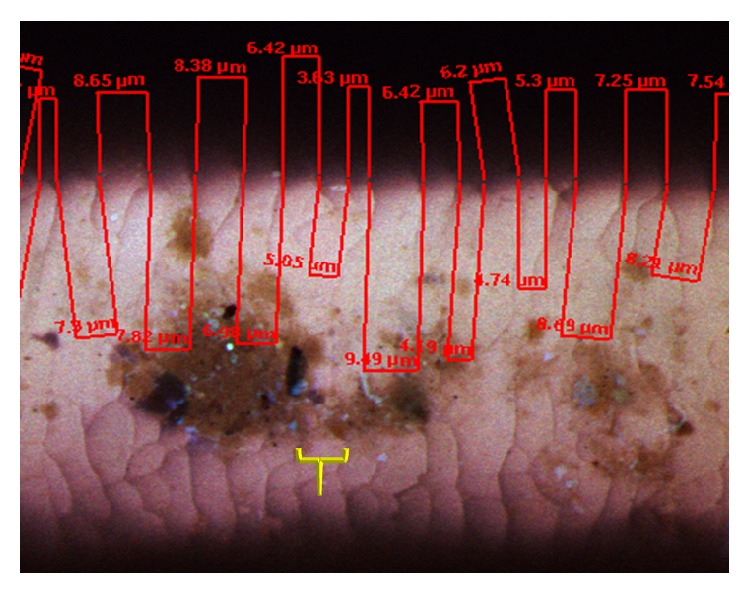
Confocal microscope image of single hair for measuring the repeat intervals (RI). The yellow bracket delineates the limits of one RI. Actual measures of some RI are shown in red. Brown patches are fungus growing on this hair.

**Figure 5:**
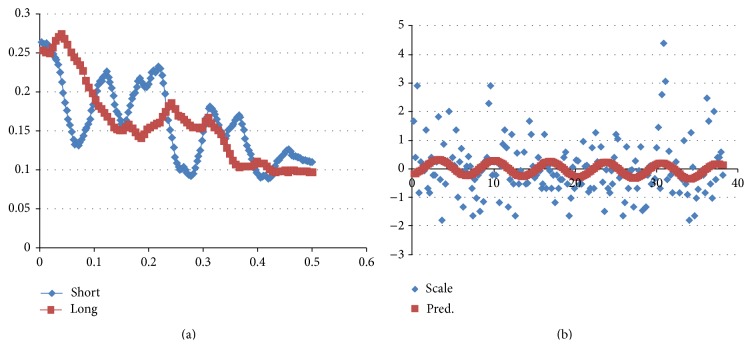
(a) Blue, control; red Zweeloo woman. Power spectra. (b) There is a high frequency peak at 0.32 radians/RI. The periodicity of this sinusoid cannot be 52 weeks, since the average long hair remnant length of 17.4 mm would represent 6.5 annual cycles with an improbable annual growth of only 3 mm per year (proof by contradiction).

**Figure 6:**
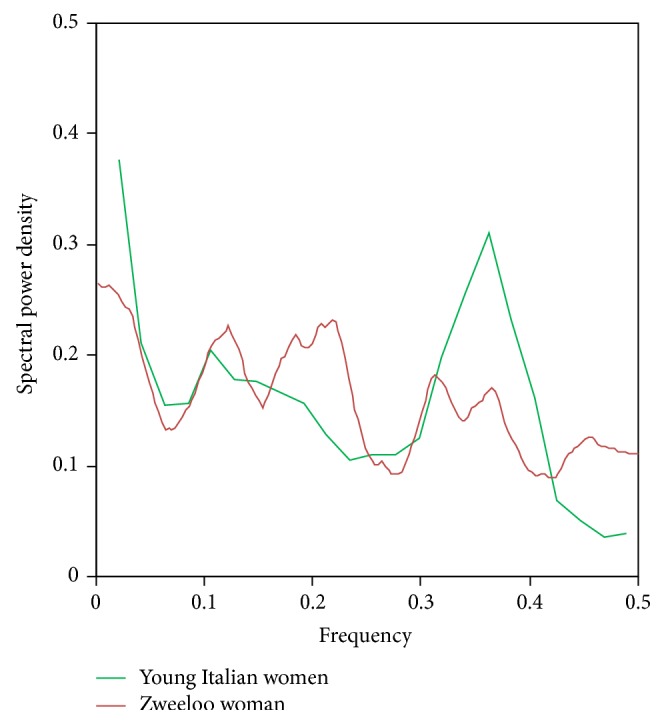
Comparative power spectra for a contemporary Italian woman's hair with normal growth rate of ~17 cm/year.

**Figure 7:**
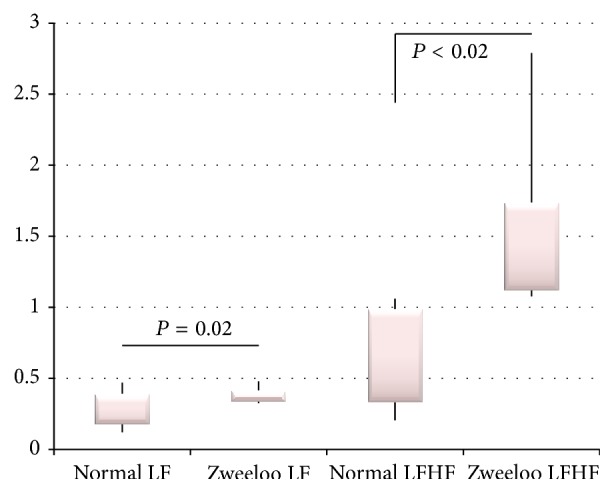
Modeled comparisons of Zweeloo woman's low frequency and LFHF ratios.

Using confocal microscopy images, we modeled the biologic rhythms by measuring the growth intervals, the widths of the repeat intervals (RI) on the hair (Figure 4) [8, 9].

Repeat intervals reflect hair growth; this in turn depends on metabolism which is affected by nutrition and environmental factors [8, 9]. Additionally, mental states in humans have overriding effects on the growth of the hair by affecting hormonal secretions and autonomic nervous system (ANS) activity [10, 11]. Therefore, using modeling of the power spectra derived from the RI's inferences about the pathophysiology and mental state can be made [12].

The low frequency/high frequency (LFHF) ratios were significantly increased (*P* < 0.02) and the low frequency (LF) variance significantly decreased (*P* = 0.02) consistent with an increased ANS sympathetic drive (part of the ANS) to metabolism and also increased heart rate and stress during life.

To validate our model of metabolism we used approximate entropy (ApEn). This technique is used in the analysis of repetitive medical data such as heart rate variability [5, 13].

*Approximate Entropy (ApEn)*. We used ApEn to measure the logarithmic likelihood that patterns of data length (*m*) that are similar remain so within a tolerance (*r*) on the next incremental (*m* + 1) comparison. In this analysis smaller values of ApEn indicate greater regularity in the data. Larger values are indicative of greater irregularities, more chaotic and more robust, systems (Figure 9).

## 4. Discussion

Bioarcheology is best described as the study of human remains from archeological excavations. This scientific endeavor deals with the examination of long dead tissues, mostly bone and hair. Therefore, life's activities such as metabolism, mood, and appearance cannot, usually, be determined from bioarcheological records. To do this suitable proxies are required using multiple methods to constrain inevitable uncertainties. Our study is based on a single specimen. Nevertheless, the multiple analytical methods and proxies used allowed us to derive sufficient data to confirm our initial hypothesis.

Here we show that the archived records of hair growth in ancient specimens provide an opportunity to model metabolism, growth, physical appearance, and behavior of individuals who lived millennia ago.

We first confirmed from witness accounts of the exhumation in 1951 that the tissues were buried in a Dutch bog. This was also supported by typical deficiencies in histological staining of the tissues caused by long immersion in the acidic bog.

### 4.1. Skin and Hair

We could not assess the preservation of the skin by ordinary histological methods because of the degradation of proteins in the specimen caused by the acidic environment of the bog. However, thick sections revealed the structure of the hairs and remnants of autonomic nerve fibers surrounding the hair bulb by their retained autofluorescence (Figure 1).

Hair styling instruments such as knifes and scissors during Roman times were made of either iron or copper [6]. The shape of the terminal end of the hair is consistent with a knife-cut rather than scissors because of its tapered appearance (Figure 2). Particles on the surface of the hair suggested that the knife may have been made of iron (Figure 3) rather than copper. Although other interpretations are also possible, notably, contamination from exhumation-instruments, these we consider unlikely in the context of this exhumation.

Using energy-dispersive X-ray spectroscopy (EDX) we also found remnants of bog-soil containing hafnium, on the surface of the hair. This rare element is commonly found in Netherland soil (P. van Gaans, personal communication).

### 4.2. Biologic Rhythms

Hair is a continuously growing tissue [11]. Like all growing tissues hair is subject to its own biologic time. The scale-like structures visible on the hair surface (Figure 4) reflect the rhythmic oscillations of growth and quiescence, the repeat intervals (RI) [9], paced by the hypothalamus, the “head ganglion” of the ANS, and master time-keeper of oscillating changes in gene expressions throughout all tissues [8, 10]. These signals, in turn, drive networks of intracellular proteins which affect the cycles of growth of the hair and other functions controlled by the ANS such as thermoregulation and especially metabolism [10]. Morphologically, the ANS signals can be identified; they correspond to the RI's of varying widths seen on microscopy of hairs (Figure 4) [8, 9].

**Figure 8:**
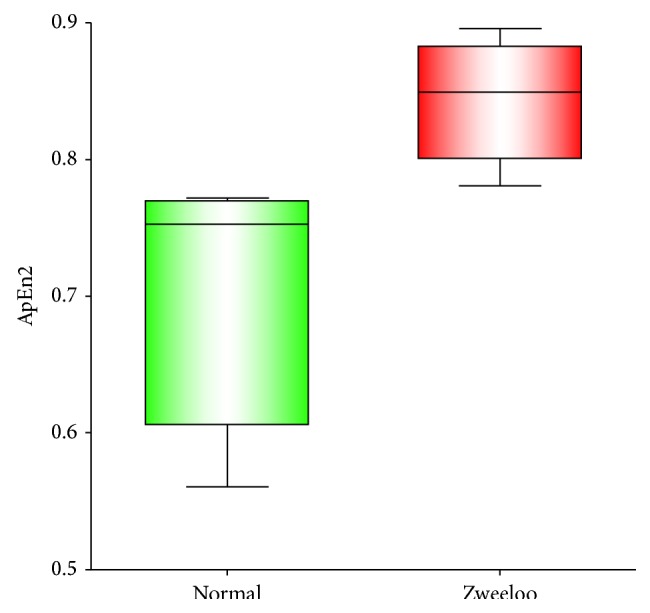
Approximate entropy (ApEn). The Low frequency/high frequency (LF/HF) ratios of the recurrent growth intervals (RI's) of normal (green) and Zweeloo woman's (red) time series.

**Figure 9:**
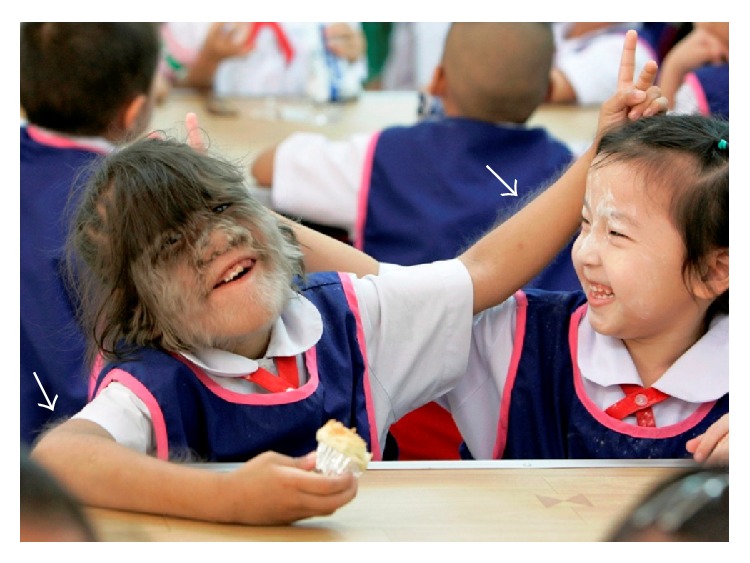
Young girl with hypertrichosis (left) and similar aged control (right). Note exuberant hair on face and both forearms (arrows) (available at http://www.mariasharapova.com/forum/).

Repetitive pattern in time series fluctuations, such as heart rate or metabolism, renders them more predictable whereas small numbers of repetitions in patterns make time series less predictable [5]. Time series with more repetitions have a small ApEn; those with fewer repetitions, therefore more chaotic patterns, have higher ApEn. We found that the ApEn of the low frequency/high frequency ratios in RI's were significantly larger in Zweeloo woman than in controls (*P* = 0.01) consistent with an enhanced sympathetic drive to metabolism (Figure 8).

The RI allowed us to determine the rate of annual hair growth [4], which for normal scalp hair is ~16 cm/year [11]. We found a surprising 153 cm/year consistent with hypertrichosis, a condition characterized by an excessive hair growth [2]. Using the lengths of her cropped hair remnants we determined that she survived for ~3–5 days after the hair cut based on her excessive hair growth rate (~153 cm/year).

Biologic rhythms in living humans are often gleaned from heart rate variability which can be analyzed statistically by spectral methods [10, 14]. Such analyses give insights into health and disease of the subjects [10, 14].

We applied the same spectral methods to the analysis of the widths of the RI's in one of the best archived materials from archeological specimens, hair. Such analyses can also yield annual cycles of growth of the hair [4].

### 4.3. Cantú Syndrome

Previous studies based on her skeletal remains established that she suffered from Léri-Weill dyschondrosteosis which consists of bony abnormalities and clinically short stature [1]. This condition in combination with hypertrichosis and cardiomegaly constitutes a recently described syndrome designated Cantú syndrome [3].

Thus using archived material together with computational modeling we deduced her unusual physical appearance due to excessive hair growth and short stature and accelerated metabolic state including mental arousal. She survived for 3–5 days after the hair cut, as determined statistically 2000 years after death and burial in a Dutch bog.

Our results confirm that a record of metabolism and behavior is archived in the spectral power of the RI's of the hair which, in turn, gives insight into life as it was millennia ago and into other aspects of Zweeloo woman's last days before her burial in a Dutch bog.

Heart rate variability in health sciences has been extensively used as indicator of impending failure of metabolism (death) and for predicting progression of disease [10, 15]. Psychosocial investigations also use heart rate as a measure of emotions and arousal states of normal subjects [15]. We used the same computational methods applied to the RI of archived hair and were able to infer her unusual appearance during life and her emotional arousal for a few days before she died 2000 years ago.

### 4.4. Hirsutism

Idiopathic hirsutism is an inherited disorder associated with unusual physical features caused by excessive hair growth [16]. In the recent past, those afflicted with this disorder may have made their living by appearing in “freak-shows” such as Julia Pastrana born in 1834 in Mexico. She was sold to a freak show manager who exhibited human oddities throughout the United States and Canada. She suffered from hypertrichosis and gingival hyperplasia giving her an ape-like appearance. Darwin mentioned her in his book* The Variation of Animal and Plants under Domestication*. Because of her unusual appearance she was also considered the “missing link” between humans and apes. In modern times, this condition is usually associated with unexplained hairiness which affects 10% of otherwise normal women in the United States; the disorder implies abnormalities in androgen actions. Though hirsutism is not lethal, in severe cases, it causes significant mental trauma and anguish [16]. Zweeloo woman's hair grew at ~10 times the normal rate of scalp hair [11] and she had excessive hair growing over her extremities (data not shown) which would have contributed to her unusual appearance.

Previous studies on Zweeloo woman [1] indicated short stature, otherwise normal health for that time in history, but failed to provide a definite clue for the reason of her unusual burial. We surmise that because of her marked hirsutism, short stature, and peculiar appearance that these physical features may have been decisive in leading to her burial in a bog.

### 4.5. Modeling

Advances in science can be made using computational modeling and validating the models with additional data. In bioarcheology and evolutionary genomics computational modeling has also recently been used [17, 18].

Here we use archived hair from the Roman period. We have validated our results in living people [12, 19] and in a variety of animals such as mammoths [20]. We also used RI's from different tissues such as teeth from humans and hominines [12] and confirmed our statistical methods in these additional growing materials.

The adoption of mathematical models successfully used in the analysis of repetitive physiological events such as power spectral analysis of heart rate variability [19] and in other repetitive physiological events, for example, growth and quiescence of growth, has numerous precedents [14]. In biology modeling has been applied to the analysis of tree ring repeat intervals and in ptilochronology [21], the biology of bird metabolism, deduced from repeat intervals found on bird feathers. In the analysis of ecological competition modeling using similar techniques is used to predict the abundance of bird species competing for food [22]. This method has also been applied to predict the size of industrial efforts necessary in producing materials such as concrete [14, 22].

Thus, power spectral analysis used in heart rate variability which yields insights into clinical states and emotional arousal of living people [15, 19] can be applied to the variation in the width of repeat intervals of growth and quiescence as evidenced in growth lines on ancient human hair. This application of a well established method promises to also broaden the scope of archeological and forensic investigations.

## 5. Conclusions

We show that valid deductions about metabolism, physical and mental states of long dead individuals can be made from archived hair by appropriate modeling.

Thus, we could derive metabolic data and evidence for emotional states of individuals who died millennia ago.

Our model could help in analyzing hair remnants in archeological remains and in forensic investigations.
